# Co-existence of IL7R high and SH2B3 low expression distinguishes a novel high-risk acute lymphoblastic leukemia with Ikaros dysfunction

**DOI:** 10.18632/oncotarget.10014

**Published:** 2016-06-14

**Authors:** Zheng Ge, Yan Gu, Lichan Xiao, Qi Han, Jianyong Li, Baoan Chen, James Yu, Yuka Imamura Kawasawa, Kimberly J. Payne, Sinisa Dovat, Chunhua Song

**Affiliations:** ^1^ Department of Hematology, Zhongda Hospital, Southeast University Medical School, Nanjing 210009, China; ^2^ Department of Hematology, The First Affiliated Hospital of Nanjing Medical University, Jiangsu Province Hospital, Nanjing 210029, China; ^3^ Department of Pediatrics, Pennsylvania State University Medical College, Hershey, PA 17033, USA; ^4^ Department of Biological Chemistry & Molecular Pharmacology, Harvard Medical School, Boston, MA 02115, USA; ^5^ Penn State Hershey Genome Sciences Facility, Penn State College of Medicine, Hershey, PA 17033, USA; ^6^ Department of Pathology and Human Anatomy, Loma Linda University, Loma Linda, CA 92350, USA

**Keywords:** IL7R, SH2B3, Ikaros, gene expression, acute lymphoblastic leukemia

## Abstract

Acute lymphoblastic leukemia (ALL) remains the leading cause of cancer-related death in children and young adults. Compared to ALL in children, adult ALL has a much lower cure rate. Therefore, it is important to understand the molecular mechanisms underlying high-risk ALL and to develop therapeutic strategies that specifically target genes or pathways in ALL. Here, we explored the IL7R and SH2B3 expression in adult ALL and found that IL7R is significantly higher and Sh2B3 lower expressed in B-ALL compared to normal bone marrow control, and the IL7R^high^SH2B3^low^ is associated with high-risk factors, and with high relapse rate and low disease-free survival rate in the patients. We also found that Ikaros deletion was associated with the IL7R^high^SH2B3^low^ expression pattern and Ikaros directly binds the IL7R and SH2B3 promoter, and suppresses *IL7R* and promotes *SH2B3* expression. On the other hand, casein kinase inhibitor, which increases Ikaros function, inhibits IL7R and stimulates SH2B3 expression in an Ikaros dependent manner. Our data indicate that IL7R^high^SH2B3^low^ expression distinguishes a novel subset of high-risk B-ALL associated with Ikaros dysfunction, and also suggest the therapeutic potential for treatment that combines casein kinase inhibitor, as an Ikaros activator, with drugs that target the IL7R signaling pathway.

## INTRODUCTION

Acute lymphoblastic leukemia (ALL), the leading cause of cancer-related death in children and young adults (aged 21–39), is less common in adults [1–4] and treatment outcomes are significantly inferior to those in children [1–3, 5, 6]. With front-line therapy, rates of complete remission have ranged between 78% and 93% in recent clinical trials. However, one third of patients with standard-risk ALL and two thirds of high-risk patients relapse, making the cure rate about 40 percent overall in adults [7–9]. The reasons underlying this age-related decline in outcome are not completely understood, but it is observed that a higher incidence of genetic alterations is associated with poor outcome, and genetic alterations such as the BCR–ABL1 fusion, are more common in adults than in children [10, 11]. Nevertheless, when compared to childhood ALL, detailed information on the genetic basis of ALL in adults is lacking. Therefore, it is important to understand the molecular mechanism underlying high-risk adult ALL and to develop therapeutic strategies that specifically target genes or pathways in ALL.

The cell surface interleukin (IL)-7 receptor-α (IL7R) is present in lymphoid progenitor cells, and is required for normal lymphocyte development [12]. IL7R forms heterodimers with IL-2Rγ (common gamma chain) or with the cytokine receptor-like factor 2 (CRLF2) [13, 14] and activates the JAK/STAT5 and the PI3K/Akt/mTOR signaling pathways [15]. IL7R mutations have been identified in malignant and nonmalignant diseases. Somatic mutations in IL7R are detected in 10% of pediatric T-ALL cases and in a few cases of pediatric B-ALL. IL7R gain-of-function mutations are reported to be oncogenic in childhood T-ALL and B-ALL [16, 17]. Overexpression of the IL-7R is also reported to have oncogenic effects [18], however, the mechanism responsible for IL-7R overexpression in ALL it is not fully understood.

The SH2B adaptor protein 3 (SH2B3), also known as lymphocyte adaptor protein (LNK), is a negative regulator of cytokine signaling and plays a critical role in the homeostasis of hematopoietic stem cells and lymphoid progenitors. Loss of function mutations in SH2B3 are reported to play an important role in oncogenesis of ALL [19, 20]. Loss of SH2B3 results in an increase in Janus kinase-signal transduction, activation of transcription signaling and lymphoid cell proliferation, which further promotes leukemia development in a mouse model of NOTCH1-induced ALL [20, 21]. These results demonstrate that SH2B3 functions as a tumor suppressor in the pathogenesis of ALL. However, it is unclear whether low SH2B3 expression is associated with clinical features in ALL.

Two of the three major signaling pathways that are perturbed in high-risk ALLs are 1) loss of function of the lymphoid transcription factors IKZF1 and PAX5, and 2) activating tyrosine kinase lesions [22, 23]. The IL7R/CRLF2 receptor and downstream JAK-STAT pathway plays a critical role in malignancy of B-ALL. SH2B3 regulates pro-B progenitor homeostasis by attenuating IL-7–stimulated JAK/STAT5 signaling via a direct interaction with phosphorylated JAK3. While SH2B3 suppresses IL-7R/JAK/STAT signaling to restrict pro-/pre-B progenitor expansion and leukemia development [20], Ikaros plays an essential role in lymphocyte development and functions as a tumor suppressor in leukemia. Ikaros dysfunction is associated with a high relapse rate and unfavorable outcome in high-risk ALL [24–26]. We reported that restoring Ikaros function by Casein Kinas II (CK2) inhibition has therapeutic efficacy against high-risk leukemia by suppression of expression of Ikaros gene targets [27–30]. Here, we determined SH2B3 and IL7R expression in adult ALL and found that SH2B3 expression is significantly lower and IL7R is significantly higher than in normal bone marrow. Furthermore, the lower SH2B3 expression is associated with high IL7R expression in B-ALL. High IL7R and low SH2B3 expression are associated with high risk factors. High IL7R and low SH2B3 (IL7R^high^SH2B3^low^) was associated with a high relapse rate and low disease-free survival in patients with B-ALL. Our studies show that Ikaros deletion is negatively correlated with SH2B3 expression but positively correlated with IL7R expression in patient samples and that Ikaros directly regulates IL7R and SH2B3 expression. CK2 inhibitor (TBB), which enhances Ikaros function, affected IL7R and SH2B3 expression in an Ikaros-dependent manner. Our findings indicate that IL7R^high^SH2B3^low^ expression distinguishes a novel subset of high risk B-ALL associated with Ikaros dysfunction. Our data also suggest the therapeutic potential of therapy that combines CK2 inhibitors to restore Ikaros function with drugs that target IL7R signaling.

## RESULTS

### IL7R high and SH2B3 low expression in adult ALL

**Figure 1:**
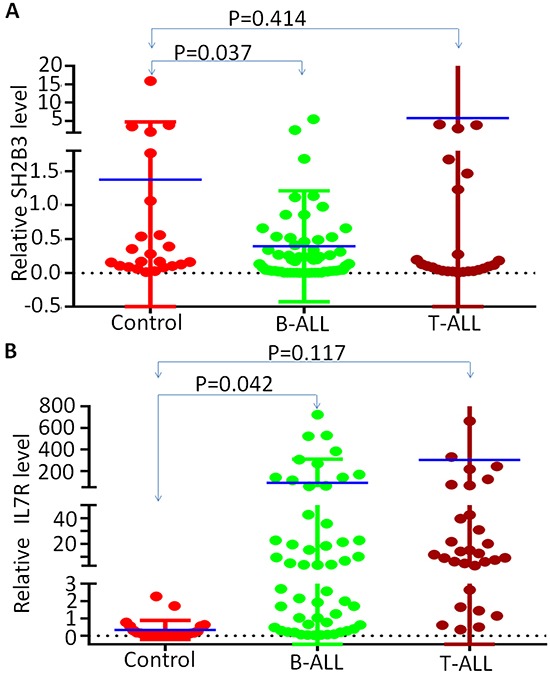
SH2B3 and IL7R expression in B-ALL patients q-PCR was performed to detect IL-7 and SH2B3 in ALL patient samples and normal BM controls. Graphed is the relative expression **A.** Comparison of *SH2B3* expression in B-ALL and T-ALL to normal BM control; **B.** Comparison of *IL7R* expression in B-ALL and T-ALL to normal BM control. Median expression is indicated and comparisons were by Mann-Whitney U test.

We assessed *IL7R* and *SH2B3* mRNA expression in 63 newly diagnosed adult B-ALL and 32 T-ALL patients. We found that, compared to normal control, SH2B3 expression is significantly lower (Figure 1A) but *IL7R* expression is significantly higher (Figure 1B) in B-ALL but not in T-ALL patients. Consistent with our data, we also observed the high expression of *IL7R* and low *SH2B3* using a reported microarray expression cohort of ALL patients (Supplementary Figure S1 and S2). We found that IL7R high expression negatively correlated with SH2B3 expression (data not shown). Frequency of high IL7R expression is significantly higher than low IL7R expression among patients with SH2B3 low expression (84.8% vs. 40.0%, P<0.0001). These data indicate that IL7R high and SH2B3 low expression (IL7R^high^SH2B3^low^) is characteristic of a subset of adult ALL.

### Association of *high IL7R and low SH2B3* expression with characteristics of adult ALL

**Figure 2:**
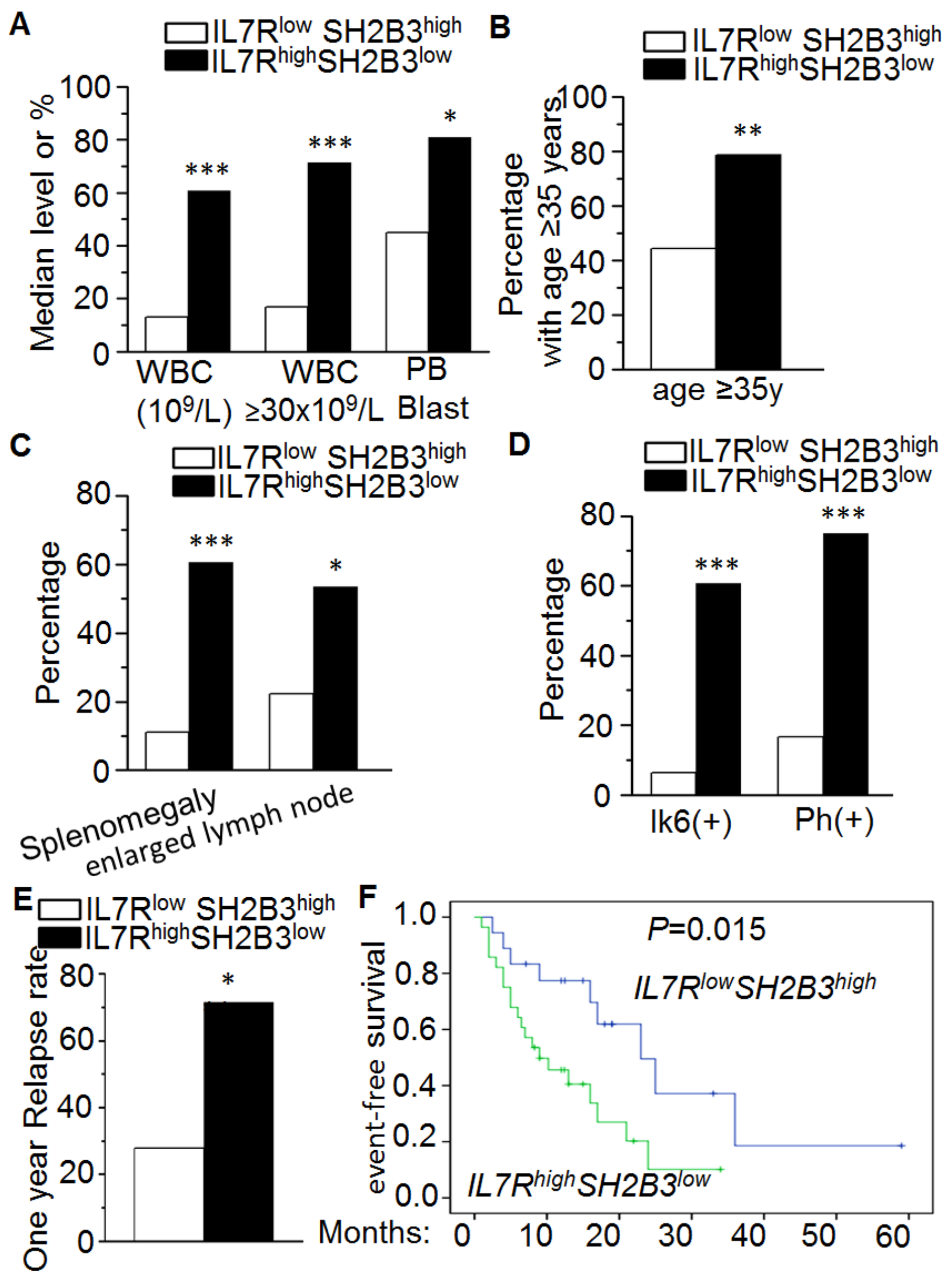
Correlation of *IL7R*^high^*SH2B3*^low^ expression *with clinical features* in ALL **A-B.** Comparison of high risk factors [high median WBC (10^9^/L), WBC≥30×10^9^/L, PB blasts] (A) and patient age ≥35 years old (B) in patients with *IL7R*^high^*SH2B3*^low^expression to those in patients with *IL7R*^low^*SH2B3*^high^expression; **C-D.** Comparison of percentage of splenomegaly or enlarged lymph node (C) and co-existence of Ik6 (+) or Ph (+) chromosome (D) in these two patients' groups. **E-F.** Comparison of one-year relapse rate (E) and event-free survival (F) in patients with *IL7R*^high^*SH2B3*^low^expression to those in patients with *IL7R*^low^*SH2B3*^high^ expression.

Patients were divided into high and low *IL7R/SH2B3* expression groups (Quartiles 3-4 vs Quartiles 1-2, respectively) and IL7R^high^SH2B3^low^ expression was correlated with clinical features in adult B-ALL (Supplementary Table S1). We found that WBC count, a poor outcome marker, was much higher in IL7R^high^SH2B3^low^ than the IL7R^low^SH2B3^high^ subgroup (median 60.7×10^9^/L vs. 12.9×10^9^/L, P=0.001). Further, WBC ≥30×10^9^/L, a specific marker for poor outcome in B-ALL, was also significantly higher in patients with IL7R^high^SH2B3^low^ (71.4 vs. 16.7, P=0.000) (Figure 2A), along with higher leukemic blasts in peripheral blood (median 81.0% vs. 45.0%, P=0.035) (Figure 2A). The percentage of the patients with age ≥35 years old, an important prognostic factor of poor outcome in adult B-ALL, was significantly higher in the IL7R^high^SH2B3^low^ than the IL7R^low^SH2B3^high^ subgroup (78.6% vs. 44.4%, P=0.018) (Figure 2B). The frequency of splenomegaly and enlarged lymph nodes, which indicates extramedullary infiltration, were found to be higher in the IL7R^high^SH2B3^low^ subgroup (60.7 vs. 11.1, P=0.001; 53.6 vs. 22.2, P=0.035, respectively) (Figure 2C). It is worth noting that we observed a significantly higher occurrence of the BCR/ABL1 fusion gene/Ph chromosome in IL7R^high^SH2B3^low^ than in the IL7R^low^SH2B3^high^ subgroup (75.0 vs. 16.7, P=0.000) (Figure 2D). We did not observe statistical differences in gender, HB, PLT, LDH, leukemic blasts in BM, myeloid markers (CD13, CD33) or the presence of complex karyotypes between the IL7R^high^SH2B3^low^ and the IL7R^low^SH2B3^high^ subgroups (P>0.05).

We also compared clinical outcomes of the patients from the two B-ALL subgroups. Patients in the IL7R^high^SH2B3^low^ subgroup did not show a higher rate of long duration (≥30 days) from initial chemotherapy to achieve CR when compared with the IL7R^low^SH2B3^high^ subgroup (32.1 vs. 16.7, P=0.451). However, we did observe a much higher 1-year relapse rate in the IL7R^high^SH2B3^low^ than in the IL7R^low^SH2B3^high^ subgroup (71.4 vs. 27.8, P=0.004) (Figure 2E). We also found that the IL7R^high^SH2B3^low^ subgroup had shorter event-free survival (EFS) than the IL7R^low^SH2B3^high^ subgroup (9 months vs. 23 months, P=0.015) (Figure 2F).

Taken together, these data show that adult B-ALL with IL7R^high^SH2B3^low^ gene expression is associated with markers of high-risk leukemia and with an unfavorable outcome. These data also distinguish patients with IL7R^high^SH2B3^low^ expression as a novel subset of high-risk leukemia.

### Ikaros binds to the promoters of the IL7R and SH2B3 and regulates their expression

**Figure 3:**
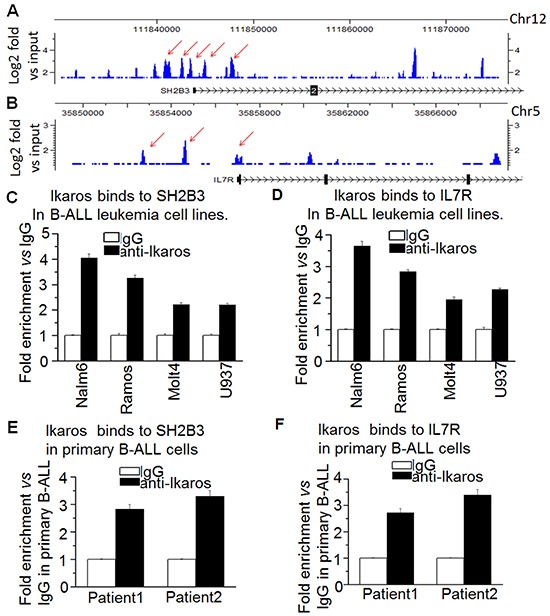
*Ikaros* binds the promoters of *SH2B3* and *IL7R* **A-B.** *Ikaros* binding peaks at the promoter of *SH2B3* (A) and *IL7R* (B). **C-D.** qChIP assay to assess Ikaros binding at the promoter of *SH2B3* (C) and *IL7R* (D) in leukemic cell lines. **E-F.** qChIP assay to assess Ikaros binding at the promoter of *SH2B3* (E) and *IL7R* (F) in primary B-ALL patients' samples.

**Figure 4:**
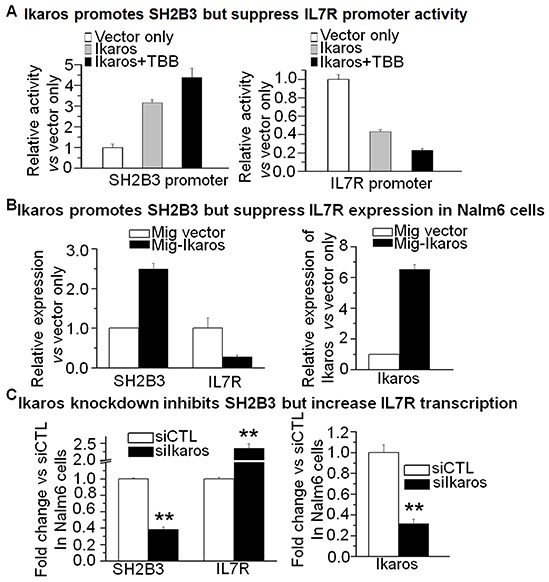
Ikaros promotes SH2B3 but suppresses IL7R transcription **A.** The promoter activity of *SH2B3* and *IL7R* promoters by luciferase reporter assay following transfection with *Ikaros* or control vector in HEK293 cells. **B.** Expression of *SH2B3* and *IL7R* in Nalm6 cells transduced with vector containing *Ikaros* as compared to control vector. **C.** Comparison of *SH2B3* and *IL7R* expression in Nalm6 cells treated with Ikaros shRNA (siIkaros) or scramble shRNA (siCTL). Gene expression is determined by RT-qPCR using total RNA isolated from the cells transfected with scramble shRNA (siCTL) or *Ikaros* shRNA (si*Ikaros*) for 2 days. compared with siCTL in C: *p <0.05, **p<0.01 compared to siCTL group.

**Figure 5:**
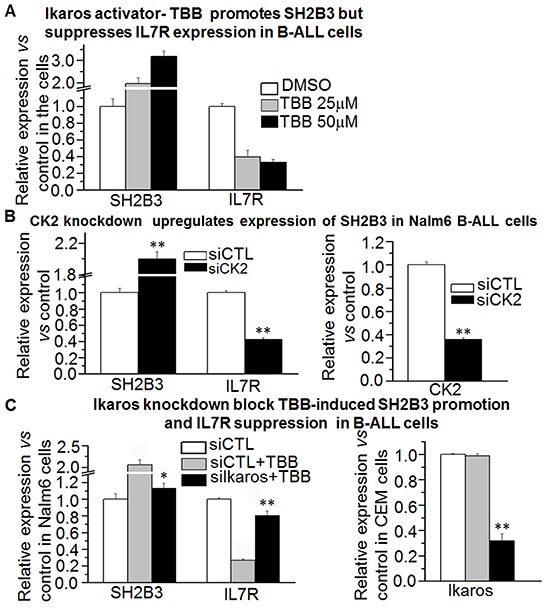
Effect of CK2 inhibitor on expression of SH2B3 and IL7R **A.** The CK2 inhibitor, TBB, promotes *SH2B3* but suppresses *IL7R* expression in B-ALL cells as assessed by q-PCR. **B.** CK2 knockdown promotes *SH2B3* but suppresses *IL7R* expression in B-ALL cells by q-PCR. **C.** Ikaros knockdown rescues the TBB-induced change in *SH2B3* and *IL7R* in B-ALL cells. Compared with siCTL+TBB in C, D and E: * P<0.05; ** P<0.01.

Our Ikaros ChIP-seq data show that Ikaros binding peaks in the promoter region of both *IL7R* and *SH2B3* in Nalm6 B-ALL cells (Figure 3A and 3B) as well as in primary B-ALL cells (Supplementary Figure S3). We identified strong Ikaros binding motifs in the promoter regions of the two genes (data not shown). The binding of Ikaros at the promoter regions of *IL7R* and *SH2B3* was further confirmed by qChIP and we found that Ikaros significantly bound to their promoter regions in Nalm6 and Ramos, malignant B cell lines (Figure 3C and 3D) and in primary cells from B-ALL patients (Figure 3E and 3F) but not in Molt 4 and U937 cells (T cell and myeloid leukemias, respectively). We further observed that Ikaros suppresses the promoter activity of *IL7R* and activates that of *SH2B3* by luciferase reporter assay (Figure 4A). These data indicate a direct effect of Ikaros on transcription of *IL7R* and *SH2B3*. Moreover, expression of *Ikaros* suppressed the *IL7R* mRNA level and increased the *SH2B3* mRNA level in Nalm6 cells (Figure 4B). Conversely, *Ikaros* knockdown induced an increase in *IL7R* expression and a decrease in *SH2B3* expression in Nalm6 cells (Figure 4C, left panel); and also Ikaros mRNA level was obviously knocked down as shown by qPCR on right panel of Figure 4. TBB (CK2 inhibitor) increased the effect of Ikaros on *IL7R* and *SH2B3* promoter activity (Figure 4A). Further treatment of Nalm6 cells with TBB suppressed *IL7R* expression and increased *SH2B3* expression in a dose-dependent manner (Figure 5A). CK2 knockdown with shRNA also suppresses IL7R but increases SH2B3 mRNA levels by qPCR (Figure 5B). Ikaros knockdown with shRNA could block the TBB-induced decrease in *IL7R* expression and increase in *SH2B3* expression (Figure 5C). These data indicate that both *IL7R* and *SH2B3* are direct Ikaros targets in ALL and that Ikaros regulates their expression.

### IL7R and SH2B3 expression in patients with an Ikaros deletion

**Figure 6:**
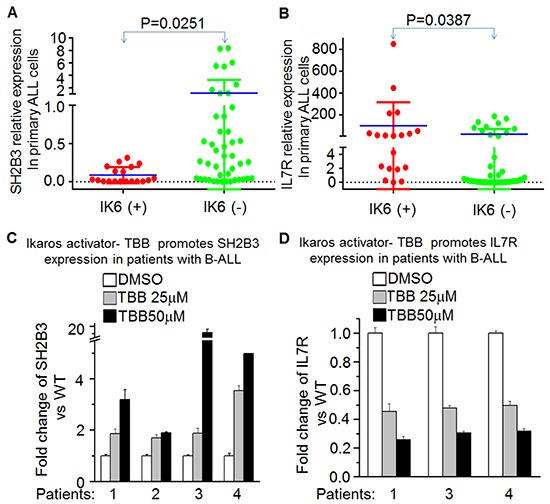
*Ikaros* deletion results in changes of *SH2B3* and *IL7R* expression in primary B-ALL cells **A-B.** comparison of *SH2B3* (A) and *IL7R* (B) in patients with or without Ikaros deletion; **C-D.** Effect of CK2 inhibitor (TBB) on expression of *SH2B3* and *IL7R* in primary B-ALL cells with TBB treatment for 2 days.

We found that Ikaros expression is positively correlated with SH2B3 expression but negatively correlated with IL7R expression in our cohort studies. Deletions that result in expression of Ikaros 6 (Ik6) are the most frequent type of Ikaros deletion. We detected Ik6 in our cohort, and further analyzed the *IL7R* or *SH2B3* expression in patients with Ikaros deletion and patients without Ikaros deletion. Our data indicate significantly higher *IL7R* expression and lower *SH2B3* expression in patients with Ikaros deletion (Ik6+), as compared to those without Ikaros deletion (Ik6-) (Figure 6A and 6B). We also found that the IL7R^high^SH2B3^low^ subgroup had a much higher frequency of Ik6+ cases (60.7 vs. 6.3, P<0.0001).

These data further support a regulatory effect of Ikaros on both *IL7R* and *SH2B3* in ALL patients and suggested that *Ikaros* deletion is one of the mechanisms for high *IL7R* and low *SH2B3* expression in the patients. Additionally, TBB can suppress *IL7R* (Figure 6E) but increase *SH2B3* expression (Figure 6F) in primary B-ALL. These data not only indicate the effect of CK2 inhibitor to increase Ikaros function as a transcriptional regulator, but also suggest that Ikaros-induced changes in *IL7R/SH2B3* expression are at least partially responsible for the success of CK2 inhibitors in ALL therapy.

### The CK2 inhibitor, TBB, promotes *SH2B3* expression through chromatin remodeling

**Figure 7:**
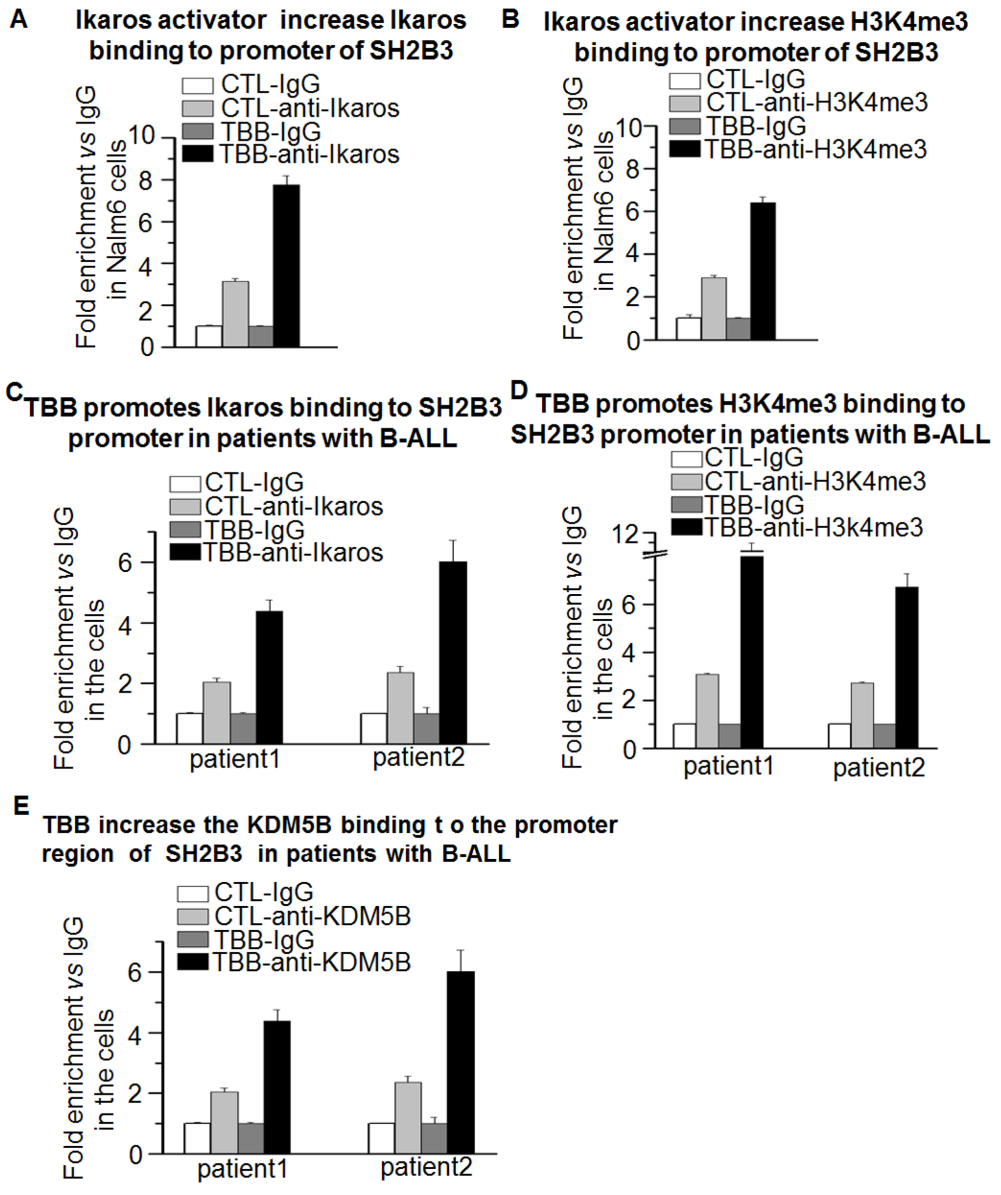
Ikaros promotes SH2B3 expression via chromatin remodeling **A-D.** The CK2 inhibitor, TBB, increases the enrichment of Ikaros (A,C) and H3K4me3 (B,D) in B-ALL cells and patients' samples. The cells were treated with TBB 25μM for 1-2 days and qChIP was performed as described in methods section. **E.** TBB increases KDM5B binding at the *SH2B3* promoter in patient samples.

CK2 inhibitor functions to increase Ikaros activity. Ikaros regulates transcription of its target genes *via* chromatin remodeling by inducing epigenetic changes around the transcriptional start site (TSS) [28]. To understand the mechanisms by which CK2 inhibitor affects Ikaros function to promote SH2B3 expression, we tested whether the activation of *SH2B3* is achieved through an epigenetic mechanism. Changes in the epigenetic markings around the TSS of *SH2B3* were measured by qChIP following TBB treatment. Results show that the TBB induces a strong binding of Ikaros (Figure 7A) and enrichment of histone H3 trimethylation at lysine 4 (H3K4me^3^) (Figure 7B) in the promoter region of *SH2B3* in Nalm6 cells, and also in primary B-ALL cells (Figure 7C and 7D). In addition, we also observed a TBB-induced increase of KDM5B binding in the promoter of the SH2B3 promoter in primary B-ALL cells (Figure 7E).

## DISCUSSION

**Figure 8:**
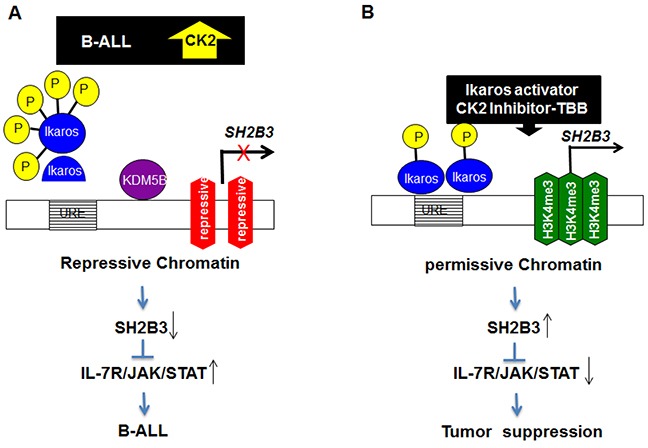
Model for the mechanism of *Ikaros* regulation of *SH2B3* expression and *IL7R/JAK/STAT* signaling

Identifying stage-specific signaling events in malignant transformation and progression and understanding the earliest stages of cellular transformation will provide critical insights in the field of leukemia. These insights will help us to decipher the mechanisms underlying high-risk ALL, delineate a new understanding of B-ALL development and point to potentially drugable pathways for intervention [31, 32]. We identified a novel subclass of high-risk B-ALL that is characterized by IL7R^high^SH2B3^low^ expression and associated with Ikaros dysfunction. Ikaros directly binds to and suppresses IL7R and stimulates SH2B3 expression in leukemic cells through chromatin remodeling as shown in the molecular model (Figure 8). Our findings indicate the collaborative effect of Ikaros dysfunction and the IL7R/JAK-STAT/SH2B3 signaling pathway on oncogenesis in high-risk B-ALL. This suggests that the use of CK2 inhibitors to restore Ikaros function combined with the inhibition of IL7R/JAK-STAT could be a potential therapy for treatment of high-risk B-ALL.

Most high-risk B-ALL-associated mutations/deletions/translocations identified to date are known or predicted to activate oncogenic cytokine receptor signaling, particularly of JAK-associated pathways [33]. We found that patients with IL7R^high^SH2B3^low^ expression have features of high-risk leukemia, unfavorable outcome and a high rate of both Ikaros deletion (60%) and BCR-ABL fusion (75.1%). It is reported that SH2B3 loss-of-function mutations co-occurs with IL7R gain-of-function mutations frequently in Ph-like ALL [22, 34]. Ph-like ALL is characterized with BCL-ABL(−), high CRLF2 rearrangement and high rate of Ikaros deletion. The characteristics of the patients that we identified are high IL7R and low SH2B3 expression, along with a high rate of BCR-ABL (+) and Ikaros deletion (+). This suggests that the patients may be different from both Ph(+) ALL and Ph-like ALL, and may account for a novel subclass of high-risk B-ALL in adult.

IL7R is highly expressed in ALL and IL7R/JAK/STAT signaling plays a critical role in oncogenesis of ALL. SH2B3, in addition to regulating normal and malignant HSC expansion via the TPO/MPL/JAK2 pathway [35–37], also plays a direct role in B cell progenitors. SH2B3 controls pro-B/pre-B homeostasis and aging by regulating IL-7–mediated JAK/STAT signaling in normal and malignant B progenitors. We identified a subclass of high-risk ALL patients characterized as IL7R^high^SH2B3^low^ which is consistent with the reported effect of IL7R and SH2B3 in the oncogenesis of B-ALL [19, 21, 34]. It is reported that SH2B3 interacts with JAK3 and IL7R activates JAK3 in B cell lineages [20]. SH2B3 is a negative regulator for IL7R–mediated JAK/SAT signaling that contributes to precursor B-ALL development. Moreover, the elevated activations of STATs such as STAT5 are downstream of IL7R stimulation in ALL [20, 38]. STAT5 is considered indispensable for maintenance of *BCR-ABL*–positive leukemia [38]. Furthermore, overexpression of oncogenic STAT5 in BM transplant models promotes B-ALL development in mice [39]. Taken together this data suggest that patients with the IL7R^high^SH2B3^low^ subclass of high-risk B-ALL will be sensitive to inhibitors of the JAK/STAT pathway. We have reported that CK2 inhibitor restores Ikaros function and shows therapeutic efficacy in high-risk B-ALL [27]. We show here that Ikaros directly binds to IL7R and SH2B3 and that the CK2 inhibitor, TBB, suppresses IL7R but promotes SH2B3 expression in an Ikaros dependent manner. These data indicate that B-ALL in these patients is likely to be sensitive to combination treatment with CK2 and JAK inhibitors.

Our previous studies have shown that Ikaros suppresses gene expression by recruiting repressive histone markers H3K9me^3^ and H3K27me^3^ via HDAC1 [27, 28]. We believe Ikaros suppression of IL7R expression is also mediated by induction of the H3K27me^3^ or H3K9me^3^ histone modifications to form repressive chromatin in the promoter region. Ikaros can also activate gene expression and the mechanism is not fully determined. We reported that Ikaros suppress KDM5B expression and increases levels of H3K4me3 histone modifications. [30]. We observed that the CK2 inhibitor, TBB, increases KDM5B binding and H3K4me^3^ histone marks at the *SH2B3* promoter suggesting that this could be a mechanism through which Ikaros activates *SH2B3* expression.

In summary, we identified a subclass of high-risk B-ALL with IL7R^high^SH2B3^low^ expression associated with Ikaros dysfunction. Our data implicate Ikaros/IL7R/SH2B3 signaling in oncogenesis of high-risk leukemia. We also found that CK2 inhibitor acts to increase transcriptional repression of IL7R and transcriptional activation of SH2B3 in an Ikaros dependent manner. This suggests the therapeutic potential of combining CK2 inhibitor with inhibitors of JAK/STAT downstream of IL7R/SH2B3 signaling.

## MATERIALS AND METHODS

### Patients and samples

BM samples were collected from 95 patients with ALL (63 B-ALL and 32 T-ALL) between June 2009 and June 2015 at the First Affiliated Hospital of Nanjing Medical University and Zhongda Hospital Southeast University Medical School. The ALL diagnosis was made according to the cytogenetic, morphologic, immunophenotypic, and molecular criteria of WHO Diagnosis and Classification of ALL (2008). Written informed consent was provided before enrollment in the study by all patients in accordance with the Declaration of Helsinki. The cohort study was also approved by the Institutional Review Board and the Ethics Committee of the Nanjing Medical University (Nanjing, China).

### Cytogenetic and molecular analyses

Conventional cytogenetic analysis was performed at the time of diagnosis using unstimulated short-term cultures according to the recommendations of the International System for Human Cytogenetic Nomenclature (ISCN). At least 20 bone marrow metaphase cells were analyzed for each sample.

Flow cytometry was performed on fresh pretreatment BM samples for immunophenotypic analyses. A cell-surface antigen was defined as positive when fluorescence intensity of at least 20% of cells exceeded fluorescence of negative control as previously described [29, 40].

### Cell culture reagents, plasmid construction, and retroviral gene transfer

Cells were incubated at 37°C in a humidified atmosphere of 5% CO_2._ Nalm6 cells have been previously described [27]. CCRF-CEM (CEM), MOLT-4 and U-937 cells were obtained from the American Type Culture Collection (ATCC, Manassas, VA). The cell lines werecultured in RPMI 1640 medium (Cellgro) supplemented with 10% fetal bovine serum (Hyclone). HEK 293T cells were cultured in DMEM (Cellgro) supplemented with 10% fetal calf serum and 1% L-glutamine (Cellgro). Primary human B-ALL cells were cultured in RPMI 1640 medium (Cellgro) supplemented with 10% fetal bovine serum (Hyclone). Cells were cultured with or without TBB and collected for total RNA isolation. Human HA-tagged *Ikaros* (*IKZF1*) retroviral construct and retroviral production was described previously [27–30].

### Luciferase assay

The pGL4.15 luciferase reporters construct for the *IL7R* and *SH2B3* promoters were constructed by insert of the IL7R promoter (−1000bp to 0bp) and SH2B3 promoter (−100 to +300bp). Transient luciferase assays were performed in HEK293T cells using Promega luciferase assay reagents and measured with a luminometer following the manufacturer's instructions. Luciferase activity was calculated as fold change relative to values obtained from pGL vector only control cells and expressed as a percentage of pcDNA 3.1-*Ikaros* transfection-induced luciferase activity versus that of pcDNA3.1 vector alone. All transfection and reporter assays were performed independently in triplicate at least three times.

### Real time-PCR

Total RNA was isolated using the RNeasy Mini Kit (QIAGEN). One μg RNA was reverse transcribed using SuperScript^TM^ First-Strand Synthesis System for RT-PCR Kit (Invitrogen). q-PCR was performed with qSTAR SYBR Master Mix (OriGene) using a StepOne Plus real-time PCR system (Applied Biosystems). Each experiment was performed in triplicate.

*IL7R* and *SH2B3* expression in patient samples was quantitated as previously described [29, 40]. The expression level of *IL7R* or *SH2B3* was normalized to 18s RNA and expressed as gene expression value of *IL7R* or *SH2B3*/18s RNA.

The qPCR for *IL7R* and *SH2B3* expression was performed in Nalm6 cells transfected with *Ikaros.* The results were normalized to those obtained with 18s RNA and presented as fold induction over vector controls. Primers: *18s RNA*, Sense:5′-GTAACCCGTTGAACCCCATT-3′, Antisense: 5′-CCATCCAATCGGTAGTAGCG-3′; *IL7R* Sense: 5′-AATGAAAAGGCCCCCAAGGTAGTTATCC-3′, Anti-sense: 5′-GTCGTTTCCGCAACAAGTCCTCTTC-3′; *SH2B3* Sense: 5′-TCACAGTGCAAGAAGGATACCAA A-3′, Anti-sense: 5′-TGAAAGCCAGCATCGTTCTTAGTC-3′.

### Quantitative chromatin immunoprecipitation (qChIP)

qChIP assays were performed by incubation of the chromatin with antibodies against *Ikaros* and normal rabbit IgG (Abcam) as a control [27–30]. Enrichment of the ChIP sample over input was evaluated by qPCR with three or more replicates, using specific primers in the promoter region of *IL7R* (forward: 5′-AGGGGA AGGGAGGGGAAGGGAAAG-3‘, reverse: 5′-CCCTT TCCTCCCCTCCCCTCCT-3‘) and *SH2B3* (forward: 5′-G GTTTTCTCTGCCTTAAACTCTGAA-3‘, reverse: 5′-GC TGCCAACAGGAAGATTTACTG-3′). The relative concentration of the qPCR product was presented as the fold change of the level of DNA in *Ikaros* samples in comparison to controls. It is considered the binding is significant if the fold enrichment versus IgG is ≥2.

### Ikaros shRNA knockdown

Nalm6 cells were transiently transfected with human *Ikaros* shRNA constructs in the GFP vector (pGFP-v-RS) (Origene) using the Neon Transfection System (Invitrogen). The 29-mer scrambled shRNA cassette in the pGFP-VRS vector was also used as a control. After transfection for 1 day, Nalm6 cells with transfection efficiency around 80% (green cells) and greater than 95% cell viability were further treated with 20μM TBB or vehicle control (0.01% DMSO) for 2 days and harvested for total RNA isolation. Knockdown of *Ikaros* was confirmed by measurement of *Ikaros* mRNA level using qPCR[27, 29]. Primers: *IKZF1*-F:5′-ggcgcggtgctcctcct-3′, *IKZF1*-R: 5′-tccgac acgccctacgaca-3′

### Statistical analysis

Patients were divided into high and low *IL7R or SH2B3* expression groups (Quartiles 3-4 vs Quartiles 1-2, respectively) as determined by SPSS 17.0.

For quantitative parameters, overall differences between the cohorts were evaluated using a Mann – Whitney U-test. For qualitative parameters, overall group differences were analyzed using a χ2 test. All statistical analyses were performed using the SPSS 17.0 and *P*<0.05 was considered statistically significant.

The experimental data are shown as the mean value with bars representing the standard error of the mean (S.E.M.). Determinations of statistical significance were performed using a Student *t*-test for comparisons of two groups or using analysis of variance (ANOVA) for comparing multiple groups. The *P*<0.05 was considered statistically significant.

## SUPPLEMENTARY FIGURES AND TABLE


