# Supplementary material for: Co-existence of IL7R high and SH2B3 low expression distinguishes a novel high-risk acute lymphoblastic leukemia with Ikaros dysfunction
Source: Oncotarget. 2016 Jun 14;7(29):46014–27. doi: 10.18632/oncotarget.10014 (PMC5216778; doi:10.18632/oncotarget.10014)
Supplement: Supplementary file 1 [file oncotarget-07-46014-s001.pdf]

Co-existence of IL7R high and SH2B3 low expression distinguishes a novel high-risk acute lymphoblastic leukemia with Ikaros dysfunction

SUPPLEMENTARY FIGURES AND TABLE

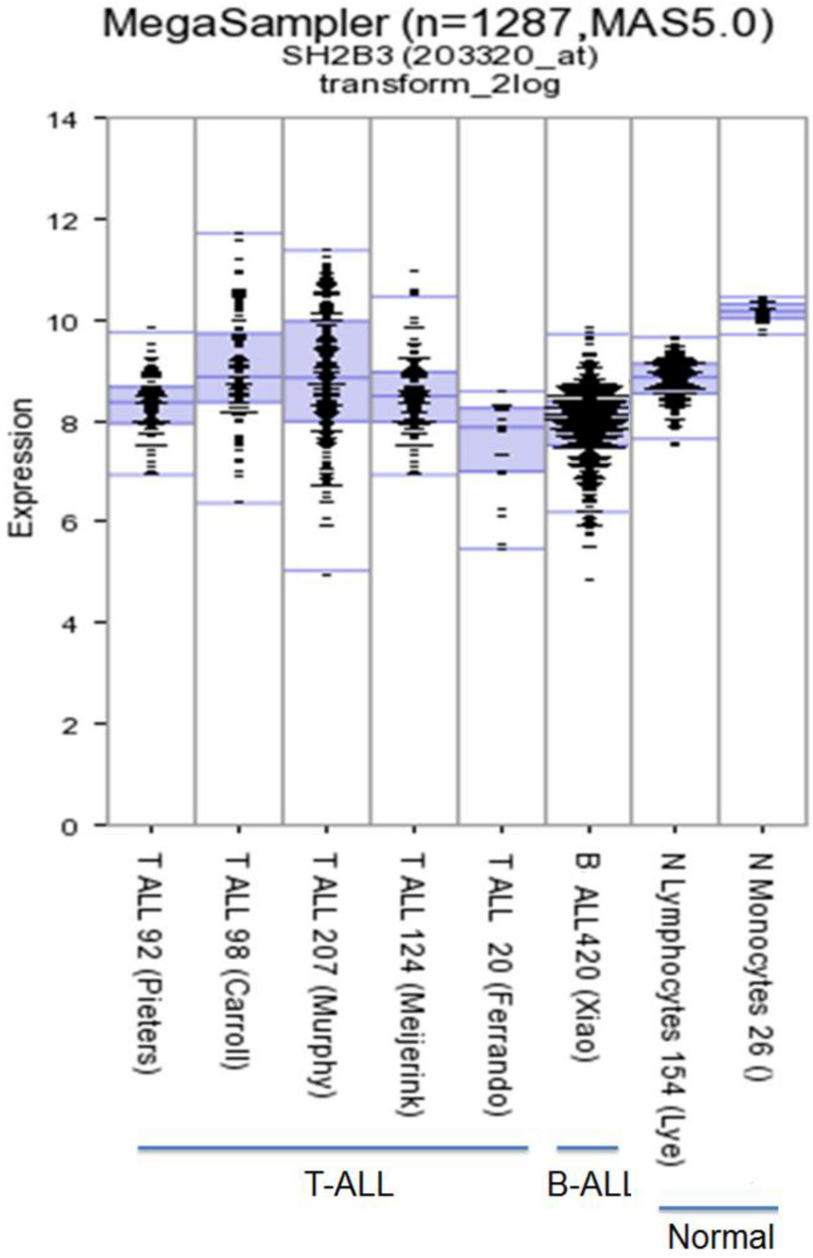

Supplementary Figure S1: SH2B3 expression in the reported ALL microarray cohort studies.

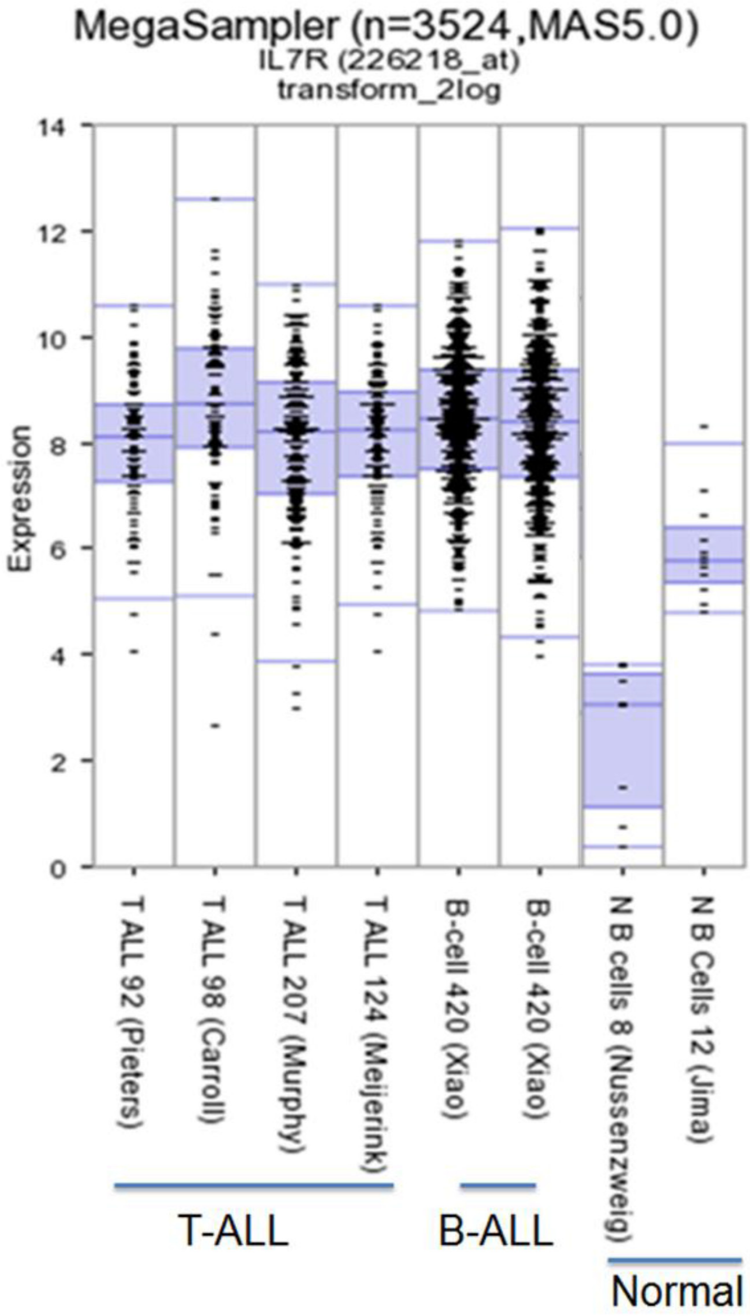

Supplementary Figure S2: IL7R expression in reported ALL microarray cohort studies.

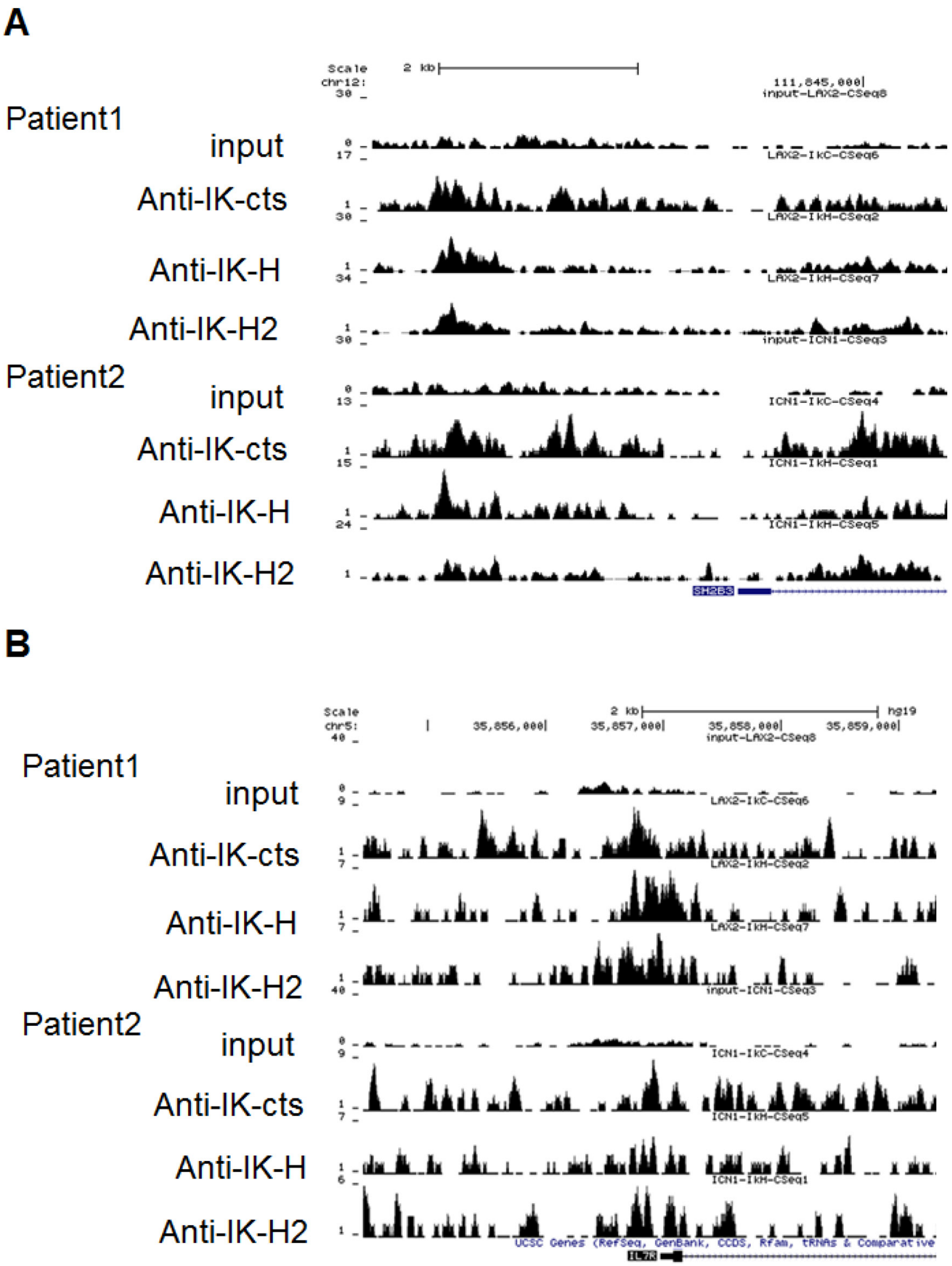

Supplementary Figure S3: Ikaros binds to the promoter of SH2B3 (A) and IL7R (B) in primary B-ALL identified by ChIP-seq.

Supplementary Table S1: Correlation of IL7R^{high}SH2B3^{low} expression with clinical features in adult B-ALL

**Characteristics**	**IL7R^{high}SH2B3^{low} (N=28)**	**IL7R^{low}SH2B3^{high} (N=18)**	***P* value**
Age (years)			
median (range)	44.0(14.0-68.0)	31.0(14.0-77.0)	0.356
≥35(years)	78.6	44.4	0.018
Sex (%)			
male	42.9	72.2	0.051
WBC, $\times 10^9$/L			
median (range)	60.7(6.7-488.0)	12.9(3.5-106.9)	0.001
≥30$\times 10^9$/L	71.4	16.7	0.000
HGB,g/L			
median (range)	90.5(51.0-150.0)	82.0(44.0-138.0)	0.348
PLT,×109/L			
median (range)	47.0(9.0-269.0)	17.0(4.0-292.0)	0.076
LDH (u/L)			
median (range)	702.0(272.0-4129.0)	714.0(175.0-7142.0)	0.917
Blasts (%)median (range)			
bone marrow	89.6(47.2-98.8)	89.6(75.0-97.2)	0.914
peripheral blood	81.0(0.0-100.0)	45.0(0.0-100.0)	0.035
Immune phenotype,%			
CD13+	45.8	41.7	1.000
CD33+	45.8	45.5	1.000
Extramedullary infiltration (%)			
liver	17.9	11.1	0.841
spleen	60.7	11.1	0.001
lymph node	53.6	22.2	0.035
Genetics (%)			
IKZF1 deletion (IK6)	60.7	6.3	0.000
BCR/ABL1 fusion gene(Ph+)	75.0	16.7	0.000
complex karyotype	13.6	25.0	0.589
Duration to achieve CR(≥30 days, %)	32.1	16.7	0.451
Relapse rate (in 1year, %)	71.4	27.8	0.004
